# Surface tension, rheology and hydrophobicity of rhizodeposits and seed mucilage influence soil water retention and hysteresis

**DOI:** 10.1007/s11104-019-03939-9

**Published:** 2019-02-02

**Authors:** M. Naveed, M. A. Ahmed, P. Benard, L. K. Brown, T. S. George, A. G. Bengough, T. Roose, N. Koebernick, P. D. Hallett

**Affiliations:** 1 School of Biological Sciences, University of Aberdeen, Aberdeen, AB24 3UU UK; 2 School of Computing and Engineering, University of West London, Ealing London, W5 5RF UK; 3 Faculty of Biology, Chemistry and Earth Sciences, University of Bayreuth, Bayreuth, Germany; 4 The James Hutton Institute, Invergowrie, Dundee, DD2 5DA UK; 5 School of Science and Engineering, University of Dundee, Dundee, DD1 4HN UK; 6 Faculty of Engineering and Environment, University of Southampton, Southampton, SO17 1BJ UK

**Keywords:** Root exudate, Seed exudate, Surface tension, Viscosity, Contact angle, Soil water retention, Hysteresis

## Abstract

**Aims:**

Rhizodeposits collected from hydroponic solutions with roots of maize and barley, and seed mucilage washed from chia, were added to soil to measure their impact on water retention and hysteresis in a sandy loam soil at a range of concentrations. We test the hypothesis that the effect of plant exudates and mucilages on hydraulic properties of soils depends on their physicochemical characteristics and origin.

**Methods:**

Surface tension and viscosity of the exudate solutions were measured using the Du Noüy ring method and a cone-plate rheometer, respectively. The contact angle of water on exudate treated soil was measured with the sessile drop method. Water retention and hysteresis were measured by equilibrating soil samples, treated with exudates and mucilages at 0.46 and 4.6 mg g^−1^ concentration, on dialysis tubing filled with polyethylene glycol (PEG) solution of known osmotic potential.

**Results:**

Surface tension decreased and viscosity increased with increasing concentration of the exudates and mucilage in solutions. Change in surface tension and viscosity was greatest for chia seed exudate and least for barley root exudate. Contact angle increased with increasing maize root and chia seed exudate concentration in soil, but not barley root. Chia seed mucilage and maize root rhizodeposits enhanced soil water retention and increased hysteresis index, whereas barley root rhizodeposits decreased soil water retention and the hysteresis effect. The impact of exudates and mucilages on soil water retention almost ceased when approaching wilting point at −1500 kPa matric potential.

**Conclusions:**

Barley rhizodeposits behaved as surfactants, drying the rhizosphere at smaller suctions. Chia seed mucilage and maize root rhizodeposits behaved as hydrogels that hold more water in the rhizosphere, but with slower rewetting and greater hysteresis.

## Introduction

Limited water supply is one of the largest impediments to food production worldwide. Increasing plant drought tolerance and improving the capacity of plants to extract water from soil are fundamentally important for future sustainable food production. Plants have a natural capacity to produce compounds that interact with soils to increase the capacity to deliver water to plants and retain water in soils (Bengough et al. 2011; Deng et al. 2015). Rhizodeposits produced by roots are polysaccharide rich mucilage from their tips, border cells sloughed off from the root cap, diffusible exudates that are lost passively, secretions as a response to environmental conditions and senescence-derived compounds (Jones et al. 2009). Seeds can have myxospermous mucilage coatings that are long-chained polysaccharides (Deng et al. 2015).

The release of a range of compounds from seeds and roots can have a plethora of effects, but in this context they could facilitate good contact with soil particles, modify water retention in the rhizosphere and control the movement of water from bulk soil to the root or seed surface. Bulk soil refers to the soil beyond the rhizosphere that generally lies at a distance greater than 0.15 cm from the surface of the root. Plant root exudates and mucilages can form polymeric gels that are capable of absorbing large volumes of water (McCully and Boyer 1997) that keep the rhizosphere hydrated. Carminati et al. (2010) showed that the water content in the rhizosphere of lupin (*Lupinus albus* L.) was greater than the bulk soil during a period of active transpiration. Based on the measured water content in the rhizosphere and bulk soil, they derived a water retention curve of the rhizosphere, which was different from that of bulk soil. Similarly, Moradi et al. (2011) observed increasing soil water contents towards the root surface in rhizosphere of chickpea (*Cicer arietinum*), white lupin (*Lupinus albus*), and maize (*Zea mays*). Kroener et al. (2014) reported an increase in water retention of sandy loam soil treated with chia seed (*Salvia hispanica* L.) mucilage at any water potential, which was validated by other studies (Ahmed et al. 2014; Deng et al. 2015). Although these studies suggest that root derived compounds increase water retention in the rhizosphere, other studies showed the opposite. Increased drying of the rhizosphere was postulated by Read et al. (2003) to be due to the smaller surface tension of root mucilages compared to water. Earlier work attributed the drop in surface tension to phospholipids (Read and Gregory 1997). Whalley et al. (2005) also reported a reduction in water retention of the rhizosphere of wheat, maize and barley compared to that of bulk soil. The importance of the surface tension of rhizodeposits is currently poorly understood, but it is likely to play an important role in rhizosphere hydrology. The opposing impacts of rhizodeposits on soil water retention could potentially be explained by variations in rhizodeposit composition among plant species and their chemical characteristics (Naveed et al. 2017). Moreover, rhizosphere soil physical properties may vary depending on the drying-wetting history (Moradi et al. 2011).

With drying rhizodeposits may impact soil water dynamics by making the rhizosphere water repellent. Rhizodeposits may coat particles with material that becomes hydrophobic when it dries beyond a critical water content. Carminati et al. (2010) showed a markedly drier lupin rhizosphere on rewetting compared to that of bulk soil. It took approximately 2 days for the rhizosphere to become wet again. Similarly, Moradi et al. (2012) found significantly greater contact angles for the rhizosphere than the bulk soil after drying, suggesting water repellency in the rhizosphere. The effect of repellency on water uptake by a root system may be complex. Repellency may provide a useful hydraulic barrier that slows water loss to dry bulk soil, especially that surrounding older root tissue. In wet soil, fresh mucilages by young roots may facilitate water uptake that compensates for any slower water uptake by older root segments (Carminati and Vetterlein 2013).

In addition to root age, plant species and environmental conditions may influence how roots influence the development of water repellency. Direct measurements of water transport by Hallett et al. (2003) observed reduced water sorptivity and increased repellency in the rhizosphere compared with bulk soil for barley, but not oil-seed rape. Zickenrott et al. (2016) demonstrated that water repellency of the rhizosphere was affected by the quantity, as well as species-dependent quality, of the rhizodeposits of *Lupinus albus*, *Vicia faba*, *Zea mays*, and *Triticum aestivum*.

Clearly the rhizosphere often has different hydrological properties to bulk soil, which will have a significant impact on how plants can capture water and potentially influence water storage in soil. Rhizodeposits can be polymeric gels that hold water, or form hydrophobic coatings on dry soil, or be surface active compounds that diminish surface tension (Brax et al. 2017; Read and Gregory 1997). To date, no study has provided concurrent measurements of all these physiochemical properties of rhizodeposits and their resulting impact on water retention.

Our study tests the hypothesis that the physicochemical characteristics and origin of rhizodeposits and seed mucilage controls their impact on the water retention characteristics of soil. We used rhizodeposits collected by hydroponics from barley and maize roots, and by washing the mucilage coating from chia seed (Naveed et al. 2017). The surface tension and viscosity were measured at a range of concentrations of rhizodeposits and seed mucilages. They were then mixed with soil and their impact on soil water repellency, water retention and hysteresis was quantified. With these data, we propose a conceptual framework showing the significance of surface tension and viscosity of rhizodeposits in modifying hydraulic properties of the rhizosphere.

## Materials and methods

### Collection of rhizodeposits and seed mucilage

The collection of rhizodeposits and seed mucilage used the same approaches as Naveed et al. (2017). For rhizodeposits this will include mucilage, secretions and border cells, as the only feasible method to collect large enough volumes to measure soil water retention impacts was hydroponics.

### Extraction of chia seed mucilage

To collect chia mucilage, 10 g of seeds were mixed with 100 g distilled water for 2 min at 50 °C with a magnetic stirrer, and then left to cool to room temperature (20 °C) for four hours (Ahmed et al. 2014). Seeds were removed from the mucilage by pushing the mixture through a 500 μm sieve using pressure applied using a syringe that was cut at the end. As reported by Naveed et al. (2017) some mucilage remained bound to seeds, but after five repeated extraction attempts about 80% of the mucilage was harvested. Ball-milling of an aliquot of chia seed mucilage was done with an aim to fragment large polymers to study the effect of chia seed mucilage after fragmentation of polymers.

### Collection of barley and maize root rhizodeposits

To collect barley (*Hordeum vulgare* L., cv. Optic) and maize (*Zea mays* L. cv. Freya) rhizodeposits, plants were grown in an aerated hydroponics system (Giles et al. 2017). Surface sterilized seeds (2% hypochlorite) were pre-germinated on 1% agar (Sigma-Aldrich, Gillingham, UK) and, when the radicals reached approximately 1 cm long (2–3 days post germination), 180 individual barley or maize plants were transferred to 60 l aerated hydroponic tanks. Plants were grown with 200 μmol quanta m^−2^ s^−1^ of light under a 14 h day and 10 h night cycle. For maize the day temperature was 25 °C and the night temperature was 22 °C. For barley the day temperature was 18 °C and the night temperature was 14 °C. The hydroponic tanks were filled with a nutrient solution (pH 5.5) containing 3 mM NH_4_Cl, 4 mM Ca(NO_3_)_2_, 4 mM KNO_3_, 1 mM KH_2_PO_4,_ 3 mM MgSO_4_ and 0.1 mM Fe-EDTA with micronutrients (6 μM MnCl_2_, 23 μM H_3_BO_3_, 0.6 μM ZnCl_2_, 1.6 μM CuSO_4_, 1.0 μM Na_2_MoO_4_ and 1.0 μM CoCl_2_). To begin with the nutrient solution was at 0.25 concentration, then changed every three days to increasing concentrations of 0.5, 0.75 and finally 1.0. After 14 days growth, either 5 barley or 3 maize plants grown in the hydroponics system were then placed in 150 ml pots containing 75 ml distilled water for 12 h to collect rhizodeposits. The liquid in the collection pots was first frozen at −20 °C and then freeze-dried to concentrate the rhizodeposits. This method to collect rhizodeposits was necessary to obtain sufficient volumes and to facilitate storage, and transport between the hydroponics system and a larger freeze-drier. However, it is limited by combining all forms of rhizodeposits together and inducing artefacts through freezing and rehydrating freeze-dried samples. Carbon and nitrogen contents of 5 replicates of each of barley and maize rhizodeposits, and chia seed mucilage were measured using a CNS elemental analyser (CE Instruments, Wigan, UK).

### Surface tension measurement of the exudates and mucilages solution

Freeze-dried barley or maize rhizodeposits, or chia seed mucilage (before and after ball-milling) were mixed into distilled water to concentrations of 0.0092, 0.092, 0.92, 2.3, 4.6 and 9.2 mg ml^−1^. Surface tension of these exudate solutions was measured at 20 °C with an Attension Sigma 701 Force Tensiometer using the Du Noüy ring method (Biolin Scientific AB, Stockholm, Sweden). This measures the force required to remove a metal ring from the surface of a liquid.

### Rheological behaviour of the exudate solutions

Freeze-dried barley or maize rhizodeposits, or chia seed mucilage (freeze-dried and freeze-dried, ball-milled) were mixed with distilled water to concentrations of 0.92, 4.6 and 9.2 mg g^−1^. These exudate solutions were then measured with a Discovery Hybrid Rheometer HR-3 (TA Instruments, New Castle, DE, USA) using the same test parameters as Naveed et al. (2017). It had a cone-plate geometry (60 mm diameter, 1^o^ angle) with a gap of 500 μm. A frequency sweep test applied increasing oscillating shear stress, with stress and displacement (shear rate) measurements taken at five points for every order of magnitude of applied stress. The normal force was initially at 0 N and restricted to <0.1 N during testing, the test temperature was 20 °C controlled with a Peltier plate and the test duration was 15 min. Each test required about 1.5 ml of exudate solution and three replicates of each concentration and exudate type were measured. The apparent viscosity data as a function of shear rate were fitted with the Carreau-Yasuda model (Carreau 1968 and Yasuda 1979) as:

1 $$ \frac{\eta -{\eta}_{\infty }}{\eta_0-{\eta}_{\infty }}={\left[1+{\left(\lambda {\gamma}^{\prime}\right)}^a\right]}^{\frac{n-1}{a}} $$

where *η*, *η*_o_ and *η*_∞_ are the apparent fluid viscosity, fluid viscosity at zero shear rate and fluid viscosity at infinite shear rate, respectively. The rheological parameters *λ* is the dimensional time constant, *γ’* is the magnitude of the shear rate, *n* is the power-law index and *a* describes the transition region between zero shear rate viscosity and the power-law region. For shear-thinning fluids, the power-law index could be as small as 0.08.

### Selection and preparation of soil

Soil was collected from 0 to 100 mm depth in Bullion Field at the James Hutton Institute (JHI), Dundee (56^o^ 27′ 39′′ N and 3^o^ 04′ 11′′ W). Barley was planted in the field at the time of sampling. This soil is classified as a Eutric Cambisol, has a sandy loam texture (clay = 16%, silt = 24%, sand = 60%), 22.5 g kg^−1^ total carbon, 1.6 g kg^−1^ total nitrogen and soil pH in CaCl_2_ of 5.48 (Naveed et al. 2017). It was air-dried and then passed through a 2 mm sieve.

### Contact angle measurements

Contact angle, CA was measured on barley or maize rhizodeposits, or chia seed mucilage (before and after ball-milling), at concentrations of 0, 0.046, 0.46, 2.3 and 4.6 mg dry exudate g^−1^ dry soil. From measurements of mucilage production from a range of species, Zickenrott et al. (2016) calculated that concentrations of 0.5 to 50 mg dry exudate g^−1^ dry soil were realistic. This was achieved by adding exudate and mucilage solutions at appropriate concentrations to bring the soil to a water content of 20 g 100 g^−1^, including a control treatment prepared by just mixing distilled water in the soil at 20 g water 100 g^−1^ soil. The control soil and soils mixed with these exudate treatments were first incubated at 4 °C for 24 h to achieve homogenization. Following this, soils were allowed to dry at 40 °C for 24 h. We measured the CA on a thin layer of these soil treatments using dry soil particles fixed on adhesive tape, according to the standard procedure described by Bachmann et al. (2003). A smooth microscope glass slide was covered with double-sided adhesive tape (TESA, type 55,733, Beiersdorf), which was pressed against the exudate-treated dry soil surface for a few seconds. The slide was then lifted up gently to remove a single layer of soil particles from the soil surface. Using a syringe, one 2 μL drop of deionized water was placed on the soil sample and the CA was determined after 30 ms contact time from the three-phase boundary line (liquid–solid–gas) using a CCD-equipped CA microscope (Drop Shape Analyzer DSA25S; KRÜSS GmbH) (Ahmed et al. 2016). The contact angle of each drop is given as the mean of the left and the right sides in the images. For each concentration of the exudates and mucilages, 3 slides were prepared, and 5 measurements per slide were carried out.

### Soil water retention and hysteresis measurements

Sieved Bullion field soil was mixed with either barley or maize rhizodeposits, or chia seed or chia seed ball-milled mucilages to achieve concentrations of 0, 0.46 and 4.6 mg dry exudate and/or mucilage g^−1^ dry soil at a water content of 20 g 100 g^−1^. These soil treatments were incubated at 4 °C for 24 h to improve homogenization, and then packed in triplicate in soil cores of 3 cm diameter and 1 cm height at 1.2 g cm^−3^ bulk density.

Soil cores were saturated overnight and water retention characteristics were measured using polyethylene glycol, PEG and dialysis tubing to equilibrate soil samples at water potentials of −10, −50, −100, −380 and − 1800 kPa. This method to control water potential has been used in other studies (Ajdari et al. 2016; Williams and Shaykewich 1969). To measure the water potential of drier soil samples, a WP4C potentiometer (METER Group, Inc. USA) was used. The osmotic potentials of different concentrations of PEG molecular weight 20,000 (MERCK-Schuchdart) solution at a constant temperature of 4 °C were determined using a WP4C potentiometer. The concentration of PEG in g 100 g^−1^ in solution was related to the osmotic potential in MPa, *ψ* by,

2 $$ \psi =\left[2.2\times {10}^{-3}\right]{\left[ PEG\right]}^2-\left[5.1\times {10}^{-3}\right]\left[ PEG\right]\ \left(\mathrm{RMSE}=0.034,\mathrm{r}2=0.99\right) $$

The PEG solution was contained within dialysis tubing, which for our tests was Spectra/Por 1 (molecular cut-off weight of 6000–8000) with a diameter of 7 cm. The ends of the tubing were sealed using medical tubing clips. To minimise evaporative losses from both the soil cores and the PEG solution, all equipment was housed in a desiccator. The soil cores were placed on top of the dialysis tubing filled with PEG of certain concentration at a desired matric potential. The soil cores were first saturated and then the drying limb of the soil water characteristic curve was measured. The wetting limb of the soil water characteristic curve was measured on samples initially equilibrated to −1800 kPa by wetting using the PEG method.

The mass of the soil cores was recorded at regular time intervals until equilibrium was reached, with no change in mass indicating equilibration (tolerance is 1 mg). Generally, 2–3 weeks were needed for equilibrating soil cores to a certain matric potential as negative as −1800 kPa. After each week of the measurements, the PEG solution and dialysis tubing were changed. Both the drying and wetting limbs of the soil water characteristic curve were conducted at a constant temperature of 4 °C to suppress exudate decomposition in soil during measurements.

The drying limb of the soil water characteristic curve was fitted with the Fredlund and Xing (1994) model, which was selected because it provides reliable fits for a wide range of soil types and matric potentials. The wetting limb of the soil water characteristic curve was fitted with a 3rd order polynomial because of the lack of an S-shaped curve. The hysteresis index was quantified between −10 to −380 kPa matric potentials by the method of Lu and Khorshidi (2015) as given in Eq. 3. This is based on the difference in water content between the drying and wetting limbs, with a hysteresis index of 0.20 indicating a 20% difference in mean water content.

3 $$ Hysteresis\ index=\frac{\sum_{i=1}^{i=n}\ \frac{w_{di}-{w}_{wi}}{w_{mi}}}{n} $$

where, *w*_di_ and *w*_wi_ are the water contents of the drying curve and wetting curve at matric potential *i*, *w*_mi_ is the average water content at matric potential *i*, and *n* is number of matric potentials over which hysteresis index was quantified.

### Statistical analysis

Contact angle, surface tension, hysteresis index and rheology data were compared using analysis of variance with type of exudate and concentration as the categorical predictors. A graphical analysis was carried out to check the absence of autocorrelation and residual normality. Tukey tests were used for *post-hoc* mean comparison.

## Results

The general characteristics of the seed mucilage and rhizodeposits used can be found in Naveed et al. (2017). Chia seeds had 0.13 ± 0.03 (mean ± SE) g g^−1^ dry seed total mucilage, but only 0.10 ± 0.02 g g^−1^ dry seed of seed mucilage could be extracted. The average freeze-dried weights of rhizodeposits collected from individual barley and maize plant were 4.1 ± 0.9 (mean ± SE) and 6.4 ± 1.7 (mean ± SE) mg individual^−1^, respectively. Total carbon contents of freeze-dried barley and maize rhizodeposits, and chia seed mucilage were 149, 166, and 407 g kg^−1^, respectively. Total nitrogen content of freeze-dried barley and maize rhizodeposits, and chia seed mucilage were 62, 33, and 11 g kg^−1^, respectively. This resulted in C/N ratios of the exudates and mucilages of 2.4 for barley root, 5.1 for maize root and 37.0 for chia seed. The pH of the aqueous exudate and mucilage solutions at 4.6 mg g^−1^ concentration was 8.9 for barley root, 9.35 for maize root and 6.7 for chia seed.

**Fig. 1:**
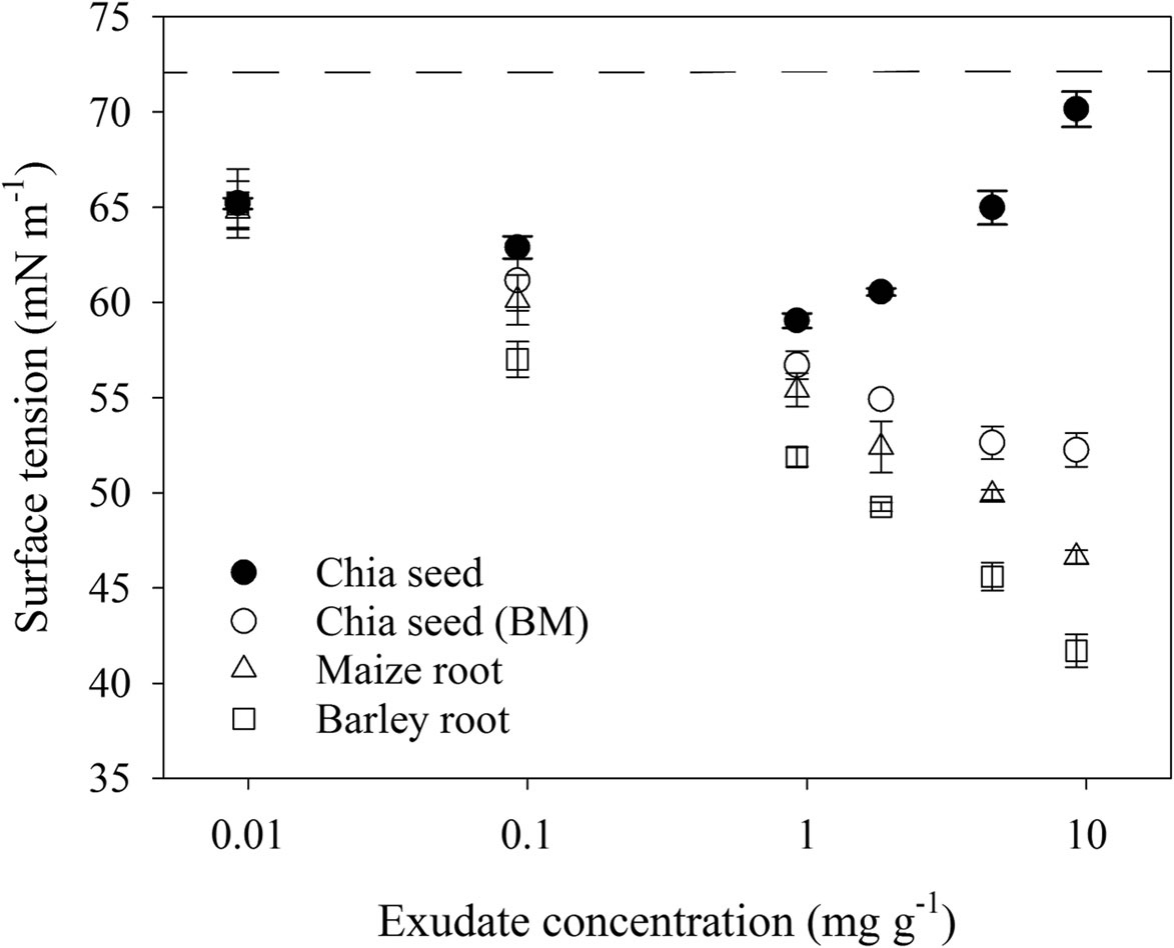
The relationship between surface tension (mean ± 1 standard error) and the concentration of the chia seed, chia seed after ball-milling (BM), maize root and barley root exudates and mucilages in water at a range of concentrations. The dashed line is the surface tension of water

Surface tension of the different plant exudate and mucilage solutions as a function of their concentration are shown in Fig. 1. With increasing exudate concentration, surface tension generally decreased. Chia seed mucilage, however, first had a decreased surface tension, followed by an increase with increasing mucilage concentration after 1 mg ml^−1^, reaching about the surface tension of water at the highest concentration of 9.2 mg ml^−1^. To test whether this was an artefact of the high viscosity of chia mucilage, ball milling was done to fragment longer chain polysaccharides. Chia seed mucilage BM had a surface tension that continued to decrease with increasing exudate concentration. With increasing concentration from 0 (pure water) to 9.2 mg ml^−1^, surface tension decreased from 72.86 mN m^−1^ (pure water) to 41.71 mN m^−1^ for barley rhizodeposits, to 46.63 mN m^−1^ for maize rhizodeposits, and to 52.26 mN m^−1^ for chia seed mucilage BM (*P* < 0.01).

**Fig. 2:**
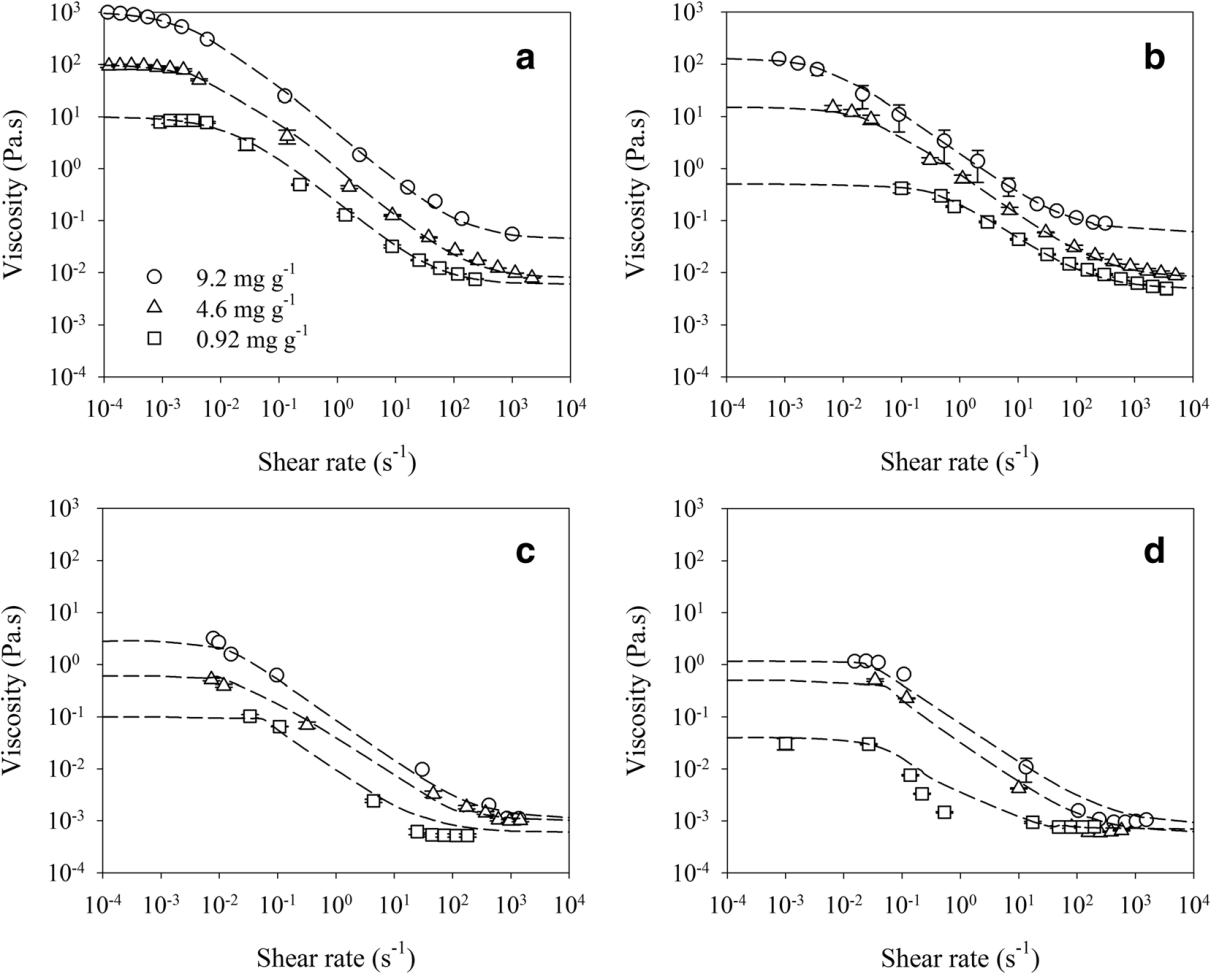
The relationship between viscosity (mean ± 1 standard error) and shear rate for different concentrations of exudates and mucilages; **a** chia seed, **b** chia seed after ball-milling (BM), **c** maize and **d** barley

Chia seed mucilage BM, maize rhizodeposits and barley rhizodeposits showed non-Newtonian behaviour as their viscosity depended on shear rate. The Carreau-Yasuda model (Eq. 1) described the viscosity as a function of shear rate data for chia seed mucilage, chia seed mucilage BM, maize rhizodeposits and barley rhizodeposits at 0.92, 4.6 and 9.2 mg ml^−1^ concentrations (Fig. 2). The model fitting parameters are provided in Table 1. The greatest viscosity at zero-shear rate was measured for chia seed mucilage, followed by chia seed mucilage BM, maize rhizodeposits and barley rhizodeposits (*P* < 0.01). Similar to this, viscosity at infinite-shear rate (asymptote) was greatest for chia seed mucilage, least for barley rhizodeposits, with maize rhizodeposits in between these extremes (*P* < 0.01). Both zero- and infinite-shear rate viscosities were decreased with decreasing concentration for chia seed mucilage, maize rhizodeposits and barley rhizodeposits (*P* < 0.01).

**Table 1:** Carreau-Yasuda model parameters obtained by fitting concentration-viscosity curves

Exudate and mucilage	Concentration	*η*_0_	*η*_inf_	a	*n*	*λ*
	mg ml^−1^	Pa.s	Pa.s	–	–	sec
Chia seed	9.2	1030	0.065	0.8	0.08	350
	4.6	95.1	0.008	0.8	0.08	150
	0.92	9.6	0.006	0.8	0.08	60
Chia seed (Ball-milled)	9.2	126.8	0.061	1	0.2	200
	4.6	12.1	0.008	1	0.2	40
	0.92	0.47	0.005	1	0.2	2.2
Maize root	9.2	2.8	0.002	1.5	0.2	80
	4.6	0.65	0.001	1.5	0.2	35
	0.92	0.11	0.0007	1.5	0.2	30
Barley root	9.2	1.16	0.0008	3	0.2	40
	4.6	0.50	0.0006	3	0.2	30
	0.92	0.05	0.0005	3	0.2	20

*η*_0_ = zero-shear viscosity, *η*_inf_ = infinite-shear viscosity

**Fig. 3:**
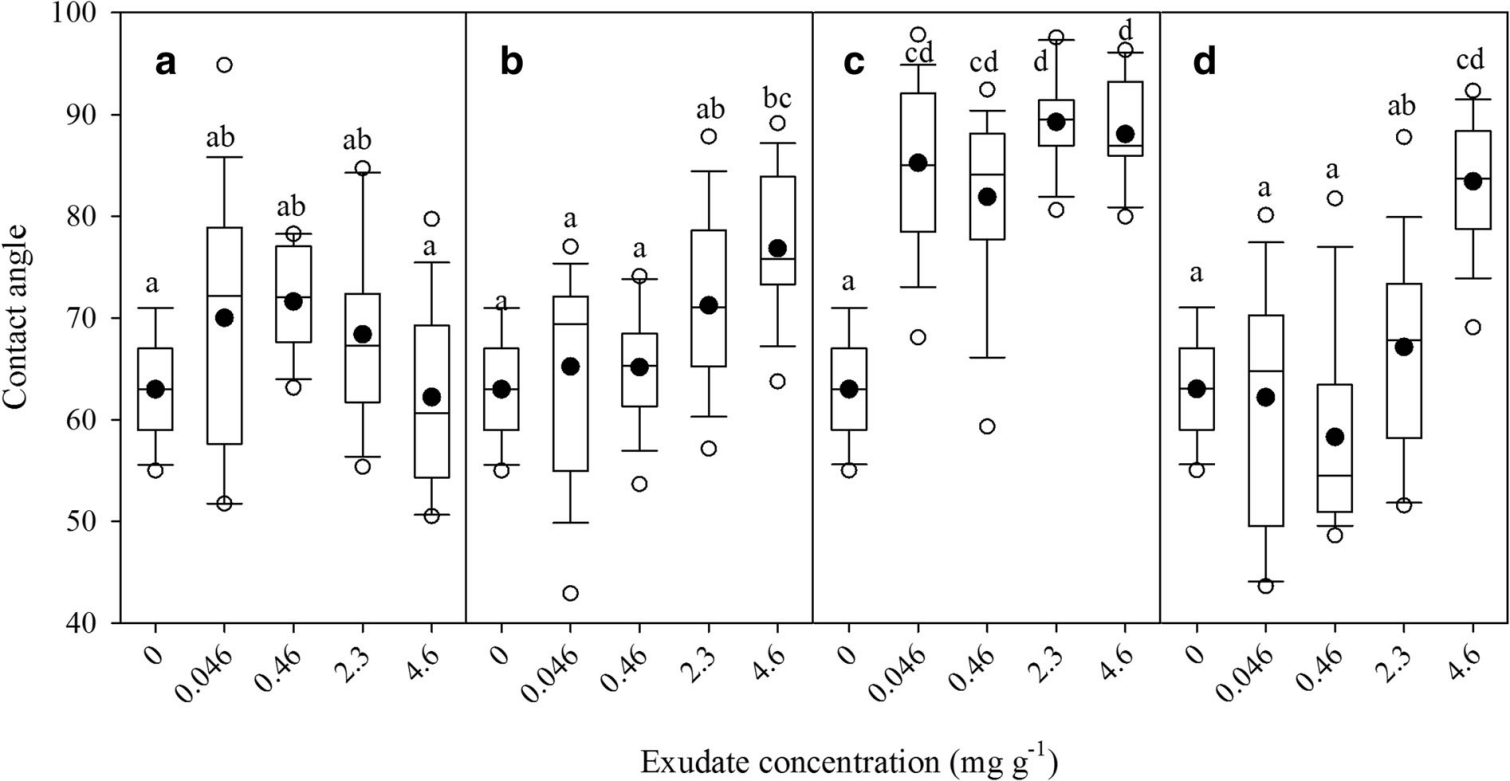
Contact angles (mean ± 1 standard error) on dried soil of **a** barley root, **b** maize root, **c** chia seed exudate after ball milling (BM) and **d** chia seed exudates and mucilages treated soil at different concentrations in water

Contact angles (a measure of soil water repellency) of water on soils amended at 0, 0.046, 0.46 and 4.6 mg g^−1^ concentrations of barley or maize rhizodeposits, or chia seed mucilage (before and after ball milling) are shown in Fig. 3. The 2-way ANOVA showed that both the source of exudate as well as their concentration in soil significantly affected contact angle, with a significant exudate-concentration interaction (Table 2). Barley rhizodeposits did not significantly affect contact angle at any of the tested concentrations. Contact angle generally increased with increasing maize rhizodeposits concentration in soil, but significant impacts were only observed at 4.6 mg g^−1^. For chia seed mucilage after ball milling, contact angle was significantly greater for all the tested concentrations compared to the untreated control. For chia seed mucilage without ball milling, contact angle was only significantly greater at 4.6 mg g^−1^ concentration compared to the control (Fig. 3).

**Table 2:** Summary results for the contact angle from Two-way ANOVA

Source	*df*	*SS*	*MS*	*F*	*P*
Exudates and mucilage	3	11,291	2763	62.6	< 0.001
Concentration	4	5361	1340	22.3	< 0.001
Exudates and mucilage.concentration	12	8448	704	11.7	< 0.001
Residual	280	16,834	60		
Total	299	41,935	140		

*df*, degree of freedom; *SS*, sum of square; *MS*, mean square

**Fig. 4:**
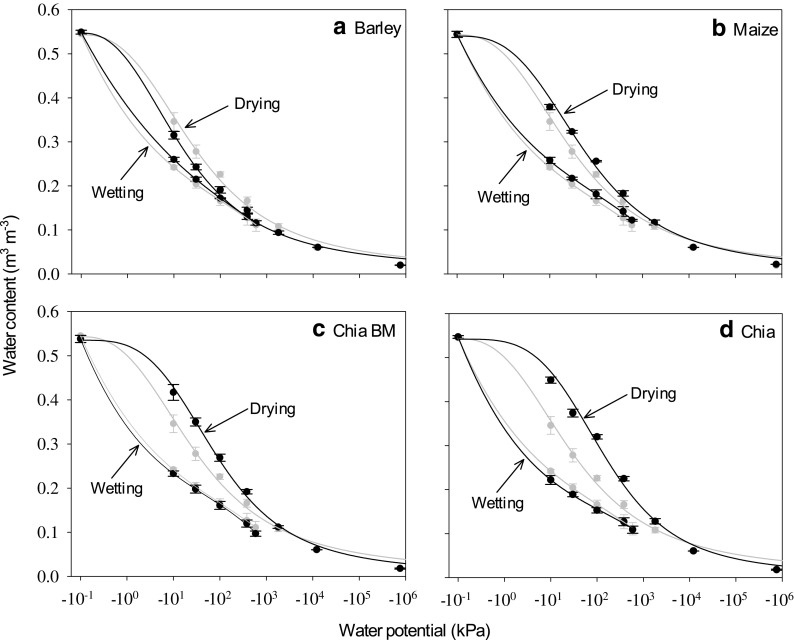
Drying and rewetting curves at a range of matric potentials for unamended soil and soil treated with exudates and mucilages at a concentration of 4.6 mg exudate g^−1^ dry soil. Grey shows the control soils that were not amended with exudate. The mean ± 1 standard error is shown. The drying curve is fitted with the Fredlund and Xing (1994) model. The wetting curve is fitted with a 3rd order polynomial

**Fig. 5:**
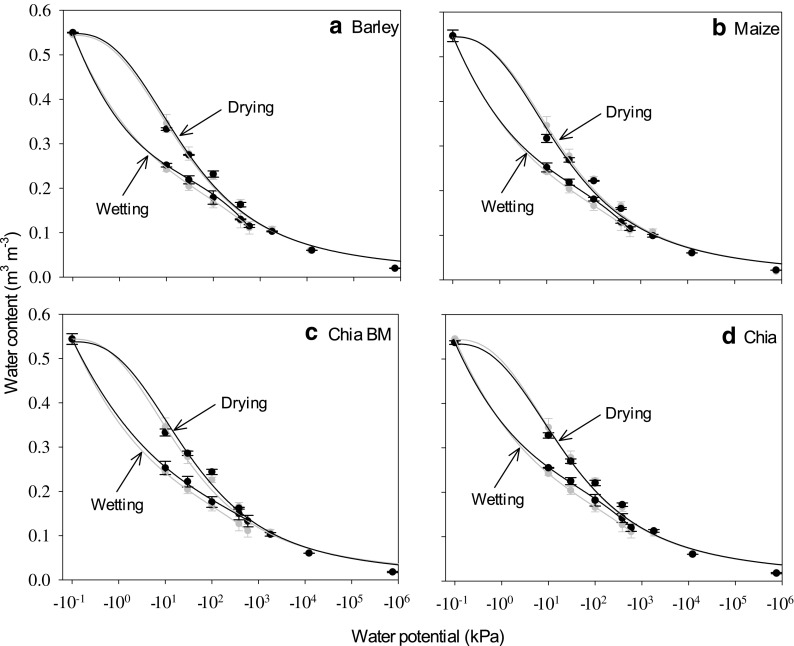
Drying and rewetting curves at a range of matric potentials for unamended soil and soil treated with exudates and mucilages at a concentration of 0.46 mg exudate g^−1^ dry soil. Grey shows the control soils that were not amended with exudates and mucilages. The mean ± 1 standard error is shown. The drying curve is fitted with the Fredlund and Xing (1994) model. The wetting curve is fitted with a 3rd order polynomial

Chia seed mucilage and maize rhizodeposits enhanced soil water retention, while barley rhizodeposits decreased soil water retention at exudate additions of 4.6 mg g^−1^ (Figs. 4 and 5). For example, at −100 kPa matric potential, soil water content was increased by 42% for chia seed mucilage without ball milling, 19% for chia seed mucilage after ball-milling and 13% for maize rhizodeposits compared to unamended soil. Barley rhizodeposits at −100 kPa water potential decreased soil water content by 15% compared to unamended soil (Fig. 4). Barley and maize rhizodeposits, and chia seed mucilage (both before and after ball milling) at a concentration of 0.46 mg g^−1^ did not have a significant effect on soil water retention compared to unamended soil (Fig. 5). The Fredlund and Xing (1994) model adequately fitted the drying limb of the soil water characteristic curves both at 0.46 and 4.6 mg g^−1^ exudate concentrations (Figs. 4 and 5). The model parameters explaining the shape of drying limb of the soil water characteristic curves are given in Table 3. A 3rd order polynomial adequately fitted the wetting limb of the soil water characteristic curves for soil treated with barley or maize rhizodeposits, or chia seed mucilage at 0.46 and 4.6 mg g^−1^ concentrations (Figs. 4 and 5). There was no appreciable effect of exudates and mucilages on wetting of soils compared to the control soil at either exudate addition concentrations. Significant effects of exudates and mucilages on the hysteresis index of soil were observed at 4.6 mg g^−1^ concentration, but not at 0.46 mg g^−1^ concentration. The smallest hysteresis index was observed for barley rhizodeposits treated soil, followed by control soil, maize rhizodeposits treated soil and chia seed mucilage treated soil (Table 4).

**Table 3:** Fredlund and Xing (1994) fitted model parameters for soil water drying curves

Exudate and mucilage	a	n	m
Exudate and mucilage concentration 4.6 mg g^−1^			
Chia seed	3.27	3.79	2.72
Chia seed (BM)	3.01	3.40	2.69
Maize root	2.87	2.93	2.67
Barley root	2.29	2.45	2.60
Unamended	2.58	2.57	2.61
Exudate and mucilage concentration 0.46 mg g^−1^			
Chia seed	2.59	2.49	2.72
Chia seed (BM)	2.73	2.61	2.81
Maize root	2.49	2.47	2.69
Barley root	2.56	2.56	2.68
Unamended	2.54	2.51	2.69

**Table 4:** Hysteresis index between matric potentials of −10 and − 380 kPa for soil treated with different exudates and mucilages

Exudate and mucilage amendment	Concentration (mg g^−1^)	Hysteresis index (−)
Unamended	0	0.26 ± 0.04bc
Barley root	0.46	0.23 ± 0.05b
Maize root	0.46	0.27 ± 0.02bc
Chia seed (BM)	0.46	0.23 ± 0.04b
Chia seed	0.46	0.21 ± 0.02b
Unamended	0	0.26 ± 0.04b
Barley root	4.6	0.11 ± 0.01a
Maize root	4.6	0.33 ± 0.04c
Chia seed (BM)	4.6	0.50 ± 0.05d
Chia seed	4.6	0.63 ± 0.04d

Different letters indicate significant difference at *p* < 0.05

## Discussion

### Surface tension of the exudate and mucilage solutions

The surface tension of soil solution is normally 5–15% less than pure water depending on organic carbon concentrations, quality of organic matter, soil pH and temperature (Anderson et al. 1995). Changes in surface tension of soil solution might have important implications for the behaviour of the soil as a whole potentially altering matric potentials, unsaturated flow rates by draining water conducting pores, solute solubilities, solute diffusion rates and gaseous transfer rates at the air-water interface. We have observed that rhizodeposits and seed mucilage solutions are even more surface active compared to that of soil solution. The greatest reduction in surface tension was observed for barley rhizodeposits (43%) followed by maize rhizodeposits (36%) and chia seed mucilage after ball milling (28%) at a concentration of 9.2 mg ml^−1^ compared to pure water. This is possibly because of the difference in chemical characteristics of the exudates and mucilages, in that barley rhizodeposits had the greatest content of organic and amino acids followed by maize rhizodeposits and chia mucilage and vice versa for sugars (Naveed et al. 2017). It is known that organic acids, such as formic acid and acetic acid, generally reduce the surface tension of water (Álvarez et al. 1997), whilst sugars, such as glucose, increase the surface tension of water and are not surface active (Shaw 1980). Surface tension for barley and maize rhizodeposits measured in this study agreed well with those reported by Read and Gregory (1997), Read et al. (2003) and LeFevre et al. (2013) for different plant rhizodeposits. Surface tension of chia seed mucilage without ball milling agreed well with surface tension of plant gums obtained from different species of *Astragalus* as reported by Balaghi et al. (2010). The increase in surface tension of chia seed mucilage solutions at concentrations greater than 1 mg ml^−1^ was likely an experimental artefact caused by the viscosity due to large polymers. The Harkins–Jordan (JW) correction factors used for the Du Noüy ring method do not consider the impact of viscosity, with other studies observing this artefact in surface tension occurring for viscous biopolymers (Lee et al. 2012). Future measurements on viscous plant exudates and mucilages may avoid this by using the drop weight method to quantify surface tension.

### Viscosity of the exudate solutions

The viscosity of a liquid is a measure of its resistance to flow. Most pure liquids and dilute solutions of low-molecular-weight compounds show Newtonian behaviour; they deform at a rate proportional to the applied stress and do not recover when the stress is removed. The viscosity of the Newtonian fluids is an absolute value that does not depend on the applied shear rate/shear stress. In contrast, solutions containing larger amounts of high-molecular-weight compounds (e.g. polysaccharides) show non-Newtonian behaviour and frequently exhibit viscoelasticity, as reported by Read and Gregory (1997) for root mucilage. When a viscoelastic material is stressed some energy is dissipated as heat during deformation, but the remainder is stored elastically. The viscosity of non-Newtonian liquids depend on the shear rate. When viscosity of the non-Newtonian liquids decreases with increasing shear rate, they are depicting shear-thinning behaviour. The exudate solutions tested in the present study showed non-Newtonian shear thinning behaviour as shown in Fig. 2. The greatest viscosity was observed for chia seed mucilage without ball milling followed by chia seed mucilage after ball milling, maize rhizodeposits and barley rhizodeposits. These were in agreement with Naveed et al. (2017) who did the same measurements on this batch of exudates and mucilages, but at only one concentration (4.6 mg g^−1^) and at a different time. The variation in viscosities between different exudates and mucilages could be attributed to polysaccharides i.e. more polysaccharide in the exudates and mucilages resulted in greater viscosities (Read and Gregory 1997; Naveed et al. 2017). Chia seed mucilage had the largest amounts of free and polysaccharide derived sugars followed by maize and barley rhizodeposits. The difference in viscosity between chia seed mucilage by ball milling was likely due to the long chain polysaccharides being crushed, decreasing the size of these molecules. The viscosity of *Capsella* sp. seed mucilage measured by Deng et al. (2013) was similar to the zero-shear viscosity of chia seed mucilage at similar concentrations in the present study. Bais et al. (2005) reported zero-shear and infinite-shear viscosities for scleroglucan (a fungal exudate) that were 10 times greater than chia seed mucilage at similar concentrations.

### Impact of exudates and mucilages on soil water repellency

The different impact of exudates and mucilages on contact angle could be explained by their chemical characteristics (Naveed et al. 2017). The >60 degree contact angle measured on unamended soils has been reported for the same soil in other studies (Feeney et al. 2006) and will be due to the levels of carbon found in the soil. Soils with less carbon and smaller contact angles may be affected more by exudates. Insignificant impacts of barley rhizodeposits on contact angle might be due to the large amount of organic acids contained in exudates. Comparatively larger amounts of sugars (polysaccharides and free) in maize rhizodeposits and chia seed mucilage could explain the significantly increased the contact angle. Chia seed mucilage made the soil extremely hydrophobic on drying. Results reflected that once the soil becomes dry the barley rhizosphere would readily rewet whereas a significant delay could occur in rewetting of the maize rhizosphere. Our findings are in line with the previous studies that observed different impacts of rhizodeposits on soil water repellency depending on species. Hallett et al. (2003) measured only a slight increase in the water repellency of the barley rhizosphere. Much greater impacts were observed for maize by Ahmed et al. (2014), who measured an increase of contact angle of water of 20 to nearly 100 degrees with increasing dry mucilage concentration from 0 to 0.075 mg cm^−2^. This can impact the uptake of water by the rhizosphere, as observed by Carminati et al. (2010) who showed that the rhizosphere of lupine remained markedly drier than the bulk soil when the samples were dried and subsequently irrigated. They found that it took approximately 2 days for the rhizosphere to become wet again. However, water drop penetration time (WDPT) tests on the same soils used here found wetting occurred within 7 s for unamended soils and 32 s for soils amended with 4.6 mg g^−1^ chia seed mucilage (unpublished). This suggests that the effects of water repellency could be short-lived and have minimal impact on water retention characteristics.

The water repellency of the rhizosphere is affected by the intrinsic chemical characteristics of rhizodeposits and the initial soil water content. Although water repellency in the rhizosphere is considered a negative impact of rhizodeposits, Carminati and Vetterlein (2013) suggested that such an effect of rhizodeposits could be considered as a plant strategy for regulating water supply. For example, fresh and hydrated rhizodeposits may facilitate water uptake of young root segments, while dry and water repellent rhizodeposits may help isolate old root segments from drier soil regions.

### Impact of exudates and mucilages on soil water characteristics

Exudates and mucilages could act both as surfactants (Whalley et al. 2005; Read et al. 2003) and hydrogels (Ahmed et al. 2014; Moradi et al. 2012) in the rhizosphere, depending on their origin and chemical characteristics. Surfactants reduce the surface tension of water, and therefore the water retention of soils is likely to decrease in the presence of surfactants (Karagunduz et al. 2001). Water stored in expanded hydrogel structures may serve as a water reservoir for plant growth, especially in regions with reduced water availability (Mazen et al. 2015; Agaba et al. 2011). Desiccation of root mucilage in soil concentrates it within smaller pores and increases adsorption to mineral surfaces (Reid and Goss 1982). The fibrous structures that are produced could increase the affinity of the mucilage to store water under drought (Albalasmeh and Ghezzehei 2014), although we found the effects of exudates and mucilages were greatest under wetter conditions. Barley rhizodeposits decreased the water retention of the soil and thus acted as a surfactant in our study. This agrees with the measured surface tension of rhizodeposits (Fig. 1), which were smaller than the other plant exudates and mucilages studied. Relatively larger amounts of organic acids and fewer free and polysaccharide derived sugars present in the barley rhizodeposits could drive this decreased surface tension (Naveed et al. 2017) observed in reduced water retention of the soil.

Read et al. (2003) also reported a reduction in water retention of soil treated with phosphatidylcholine (lecithin), chemically similar to the phospholipid surfactants identified in maize, lupine and wheat rhizodeposits. In direct measurements of the water retention characteristics of rhizosphere soil, Whalley et al. (2005) reported that the rhizospheres of both maize and barley tended to be drier at a given matric potential than bulk soil. This does not agree with our observation of increased soil water retention for soils amended with maize root rhizodeposits and chia seed mucilage. However, Whalley et al. (2005) harvested rhizosphere soil from growing plants where microbial activity may decompose and alter the properties of rhizodeposits. We intentionally suppressed microbial activity by conducting measurements at 4 °C. Our earlier research found that a measured increased viscosity of soils amended with maize rhizodeposits diminished considerably following microbial decomposition, suggesting fewer long-chain polysaccharides (Naveed et al. 2017). It is likely that the influence of rhizodeposits acting as mucilaginous hydrogels diminishes over time, so these will have greater impact at a growing root tip where water uptake is most active than in older root segments.

The mucilaginous (hydrogel) impact of chia seed mucilage appears to more than outweigh the influence of decreased surface tension (Fig. 1). Further, the water retention of the soil was greatly enhanced by chia seed mucilage before ball milling compared to that after ball milling. This signifies the role of large polysaccharides in soil water retention (Brax et al. 2017). The increase in soil water retention by maize rhizodeposits and chia seed mucilage can also be explained by the relatively greater amount of sugars (polysaccharides-derived and free) contained in these exudates and mucilage compared to that of barley rhizodeposits (Naveed et al. 2017). Supporting this, Carminati et al. (2010) showed that the water content in the rhizosphere of lupine (*Lupinus albus* L.) was greater than in the bulk soil during a period of active transpiration. Moradi et al. (2012) also observed increasing soil water content towards the root surface for chickpea (*Cicer arietinum*), white lupine (*Lupinus albus*) and maize (*Zea mays*). Similar to the present study, Ahmed et al. (2014) and Kroener et al. (2014) reported a large increase in soil water retention by chia seed mucilage. Like chia seed mucilage, *Capsella bursa-pastoris* L. seed mucilage also increased soil water retention due to its hydrogel nature (Deng et al. 2015). This earlier study used the same soil and packing conditions used in the current investigation, but measured water retention characteristics with conventional suction table and pressure plate methods. The treatments not amended with exudate or mucilage that formed the controls in each experiment had very good agreement, suggesting that the PEG approach was effective at equilibrating soil water potential.

It was surprising to find no apparent differences in the wetting limbs of the water retention curves between the control, barley and maize rhizodeposits, and chia seed mucilage treated soils (Figs. 4 and 5). This reflected the importance of the initial soil water content to the development of water repellency. Our most negative water potential of −1800 kPa is drier than the permanent wilting point, and retained 0.105 m^3^ m^−3^ water content. This is in contrast to the air-dried soils where significant soil water repellency was observed for maize rhizodeposits and chia seed mucilage treatments. This suggests that water repellency induced by the exudates and mucilages in the rhizosphere is only of concern when soil dries beyond the critical limit, as may happen in the surface layers of soil during extended dry periods. Zeppenfeld et al. (2017) suggested that this may provide a competitive advantage at the ecosystem level by making the topsoil hydrophobic, so deep-rooted plants avoid competition with shallow-rooted plants. The variation in hysteresis index for different exudate treated soils (Table 4) was therefore primarily because of the difference in soil water retention during drying of exudate treated soils.

### Limitations of the experimental approach

A hydroponics based harvesting method was used to obtain sufficient quantities of rhizodeposits for our experiments. This meant that different components of rhizodeposits were not isolated and the hydrated conditions would influence their composition. The characteristics of rhizodeposits may differ in the soil environment as well as under different stresses (Hinsinger et al. 2009). For instance, we found the rhizodeposits to be alkaline, as observed by Pojasok and Kay (1990) for rhizodeposits in sand. This could be due to the secretion of anions (Hinsinger et al. 2009) that our hydroponic system would not buffer like soil. Nitrate fertiliser was also used, which other studies have observed to increase rhizodeposit pH in soil (Gahoonia et al. 1992).

We have measured surface tension, viscosity and pH of the exudates and mucilages of different cultivars of barley and maize collected using the hydroponic method. As the results were similar between different cultivars of the same species (data are not provided in the manuscript), we did not pursue further physical testing of cultivar specific impacts. Through the use of small-scale testing approaches, such as those developed by Naveed et al. (2018), and non-invasive imaging of rhizodeposit:soil interactions in soils (Brax et al. 2017; Holz et al. 2018), there is scope to test the combined impacts of cultivars and environmental conditions on soil physical changes by rhizodeposits further.

Albalasmeh and Ghezzehei (2014) discussed several studies that found mucilage production by roots to be accentuated in xeric environments as an evolutionary mechanism to decrease water stress to plants. There is ample scope for future research on individual components of rhizodeposits collected under different environmental stresses, but a challenge remains in collecting sufficient quantities. New rhizodeposit harvesting methods can help to some extent (Zickenrott et al. 2016), which could remove artefacts such as osmotic shocks inducing plasmolysis that may have accentuated exudate harvesting with hydroponics.

### Consequences of exudates and mucilages for plant water uptake and function

Depending on origin and chemical characteristics, we found that plant exudates and mucilage could increase or decrease water retention of soil at their surfaces compared to bulk soil. These contrasting roles of the exudates and mucilages have their own advantages and disadvantages. Enhanced soil water retention by exudates and mucilages, as observed in the present study for maize rhizodeposits and chia seed mucilage, could offer an advantage to plants in the water scarce areas as protection against drought: An increase in water retention of the rhizosphere or soil surrounding a germinating seed, especially when the soil is dry, may limit the drop in unsaturated hydraulic conductivity by maintaining the hydraulic contact between soil and roots (Carminati et al. 2011, 2016; Ahmed et al. 2014) or seeds (Deng et al. 2015). On or near saturation of rhizosphere and subsequent hydration of such exudates and mucilage, saturated water flow would decrease possibly because of pore clogging by viscous nature of exudates and mucilage (Kroener et al. 2014, 2016). In contrast, the reduction in soil water retention by surfactant natured exudates and mucilages, such as barley rhizodeposits in the present study, may initially help roots to extract water more easily from the fine pores (Passioura 1988). Smaller soil water contents in the rhizosphere compared to bulk soil increases air-filled porosity near to roots or germinating seeds. This might be important where soil would be more prone to poor aeration, albeit at the expense of decreased unsaturated hydraulic conductivity (Carminati et al. 2016; Dunbabin et al. 2006). Despite these studies and speculations, the impact of different types of exudates and mucilages on water flow from bulk soil through the rhizosphere to the plant roots warrants further studies to comprehensively understand root water uptake.

**Fig. 6:**
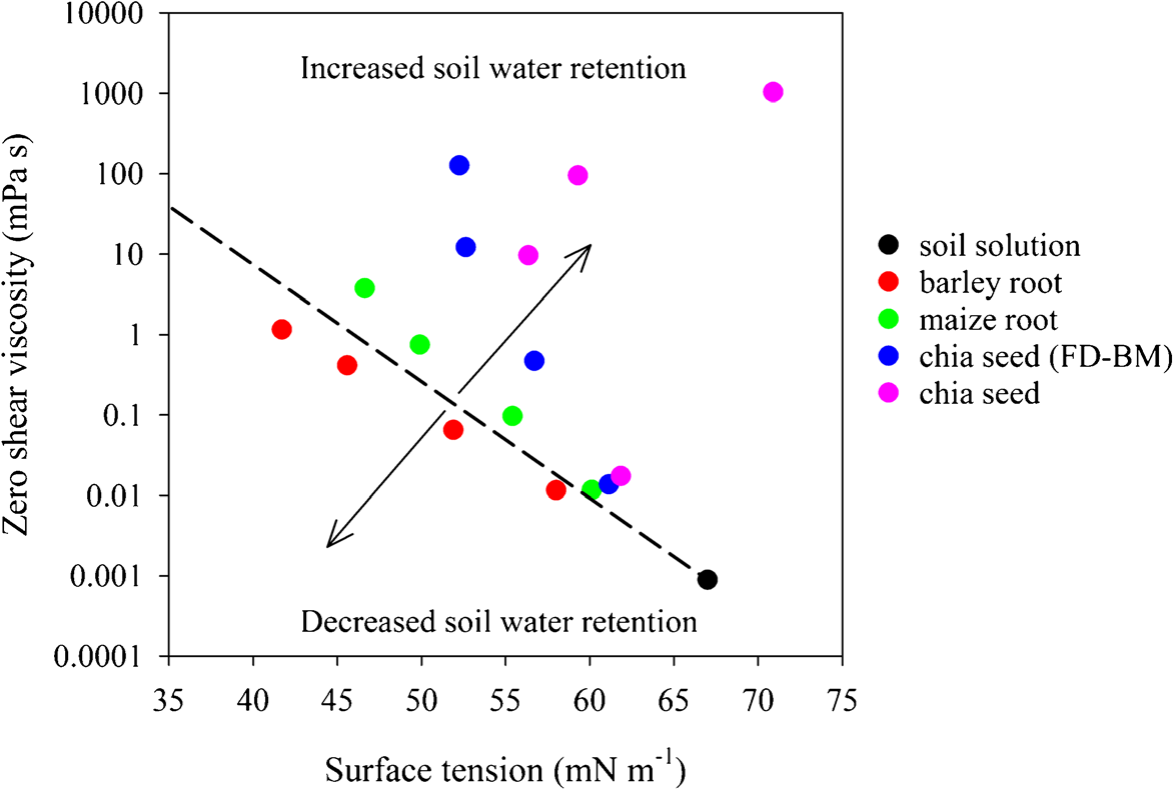
Conceptual framework showing the relative significance of surface tension and viscosity of the exudates and mucilages in soil to water retention and hysteresis. Viscosity provides an indirect measurement of long-chain polymers that may act as a hydrogel. The increase or decrease in water retention was observed from the drying limbs in Figs. 4 and 5, where only barley caused a decrease. The Dashed line is a hypothetical cut-off represents the transition between compounds that have a net effect of acting like a surfactant versus a hydrogel

Plant root exudates and mucilages have the capacity to modify both surface tension and viscosity of soil solution in the rhizosphere (Figs. 1 and 2). Generally, an increase in viscosity was coupled with a decrease in surface tension of soil solution in the rhizosphere. These modifications in the rhizosphere could impact soil-plant-water relations. Viscosity is related to the amount of long-chain polymers in the exudates and mucilages (Naveed et al. 2017) so it provides an indirect measurement of their capacity to act as hydrogels. Similarly, a decrease in surface tension of soil solution by root exudates and mucilages would tend to decrease soil water retention. In Fig. 6 we are speculating the possible scenarios of soil water retention in the rhizosphere based on surface tension and viscosity of the soil solution. If surface tension and viscosity of the soil solution lies close to hypothetical cut-off indicated by the dotted line, the soil water retention in the rhizosphere would be quite similar to that of bulk soil. As we move above the dotted line, either because of an increase in viscosity or surface tension, the soil water retention of the rhizosphere would be greater compared to that of the bulk soil. This has been measured in the case of maize root and chia seed exudates and mucilages in the present study. Similarly, if the intersection of viscosity and surface tension lies below the dotted line, the soil water retention of the rhizosphere would be less compared to that of bulk soil. This conceptual framework is based on a few data points that were measured in this study, thus future studies should aim to test the hypotheses. Further the impact of surface tension and viscosity of exudates and mucilages on soil water retention as set out in this conceptual framework also depends on the matric potential.

This research provides contrasting evidence of the influence of plant exudates and mucilages on soil water retention characteristics, which are driven by the physicochemical properties of the exudates and mucilages. As exudates and mucilages perform many functions in soil, beyond physical modification, it would be interesting to explore evolutionary drivers for differences between different plant species and possibly crop cultivars. The persistence of the impacts in relation to root age and environmental conditions remains poorly understood, but it is vital to understand how entire root systems extract water from soil. Over time rhizodeposits are decomposed, so the surfactant properties found for barley could be replaced by hydrogel properties of microbial by-products (Naveed et al. 2017). This could ultimately help to select root traits with a greater ability to tolerate drought or aeration stresses in soils.

## Conclusions

The large impact of plant exudates and mucilages on water retention characteristics can be explained by differences in surface tension, contact angle and viscosity between exudates and mucilages of different origin. These properties may be driven by the relative amounts of organic acids and sugars (free and polysaccharide derived) in the exudates and mucilages. Barley rhizodeposits, which had the lowest surface tension, contact angle and viscosity, caused soils to hold less water at a given water potential. Chia seed mucilage had the greatest surface tension, contact angle and viscosity, which caused soils to hold more water at a given water potential. Maize rhizodeposits fell in between. Whereas the drying limbs of the water retention characteristics were affected significantly by amendments with different exudates and mucilages, the wetting limbs were very similar to control soils with no added exudates and mucilages. This was unexpected and suggests that the driest point in our study (−1800 kPa water potential) was too wet to impart water repellency in this soil. Pore clogging by exudates and mucilages would be expected to decrease the wetting of soil as well, but perhaps this was offset by the water held in the exudate.

Exudates and mucilages may have important effects on soil-plant-water relations that can be explained by the origin and physico-chemical characteristics of the exudates and mucilages. This knowledge needs to be extended to understand how whole plant root systems can extract water from soil depending on exudate properties, soil conditions and decomposition.
